# Managing macular hole associated with acute inflammatory Vogt-Koyanagi-Harada syndrome

**DOI:** 10.1186/s40942-015-0020-9

**Published:** 2015-11-03

**Authors:** Rodrigo M. Navarro, Gisele C. Guizilini, Leonardo M. Machado, Mauricio Maia

**Affiliations:** 1 Vitreoretinal Diseases Unit, Brazilian Institute of Fighting Against Blindness, São Paulo, Brazil; 2 Vitreoretinal Diseases Unit, Federal University of São Paulo, Vision Institute, São Paulo, Brazil

**Keywords:** Vogt-Koyanagi-Harada syndrome, Macular hole, Pars plana vitrectomy

## Abstract

We report a 24-year-old man with Vogt-Koyanagi-Harada (VKH) syndrome who developed a macular hole (MH) during the acute inflammatory stage. Spontaneous resolution was unlikely because of the MH dimensions and absence of vitreous adherence. The patient underwent pars plana vitrectomy (PPV) and internal limiting membrane peeling during the acute stage followed by retinopexy with octafluoropropane injection and prone positioning for 5 days. The MH closed and the best-corrected visual acuity (BCVA) improved from 20/400 to 20/40. Prompt surgical intervention may be an alternative for treating MHs and obtaining visual recovery in special cases even in the acute inflammatory stage.

## Background

Vogt-Koyanagi-Harada (VKH) syndrome, which affects the uveal tissue [1, 2], is characterized by neurologic manifestations and exudative retinal detachment [3]. We report a young man with VKH syndrome who developed a macular hole (MH) and underwent pars plana vitrectomy (PPV), internal limiting membrane (ILM) peeling, octafluoropropane (C_3_F_8_) injection, and postoperative prone positioning during the acute inflammatory disease stage. To the best of our knowledge, this is the first report of a successful surgical procedure in this clinical scenario.

## Consent

This report was conducted according to the Research Guidelines of the Association for Research in Vision and Ophthalmology and the tenets of the Declaration of Helsinki. Written informed consent was obtained from the patient for publication of this Case report and any accompanying images. A copy of the written consent is available for review by the Editor-in-Chief of this journal.

## Case presentation

A 24-year-old man was referred to us with bilaterally decreased best-corrected visual acuity (BCVA) [right eye (OD), 20/200; left eye (OS), 20/400]. Biomicroscopy showed panuveitis and a serous retinal detachment. After treatment with oral prednisone (2 mg/kg/day), topical prednisone (10 mg/ml) hourly, and betaxolol (0.5 %, twice a day), the BCVA increased to 20/30 OD and 20/60 OS. He stopped treatment and after 5 months presented with worsening of the BCVA OS. Examination showed a subcapsular cataract in this eye. The patient underwent phacoemulsification OS and implantation of a foldable intraocular lens (Acrysof IQ, Alcon, Fort Worth, TX). The BCVA improved to 20/30 OD and 20/40 OS. A combination of 100 mg of oral prednisone and 5 mg/kg of cyclosporine was prescribed (both once a day).

After 3 months, the patient reported worsening of his bilateral BCVA, with hand motions OD. Indirect ophthalmoscopy, fluorescein angiography (FA) and optical coherence tomography (OCT) showed papillitis, vitreous hemorrhage, and proliferative vitreoretinopathy (PVR) due to a combined exudative/rhegmatogenic retinal detachment. Four days after pulse therapy (methylprednisolone 1 g/day for 3 days), his BCVA was counting fingers (CF) OD; fundus biomicroscopy showed worsening of the vitreous hemorrhage. PPV was carried out, with bimanual membrane dissection, endophotocoagulation and silicone oil injection (Oxane 1300, Bausch & Lomb, UK). BCVA OD improved to 20/80 after PPV.

**Fig. 1:**
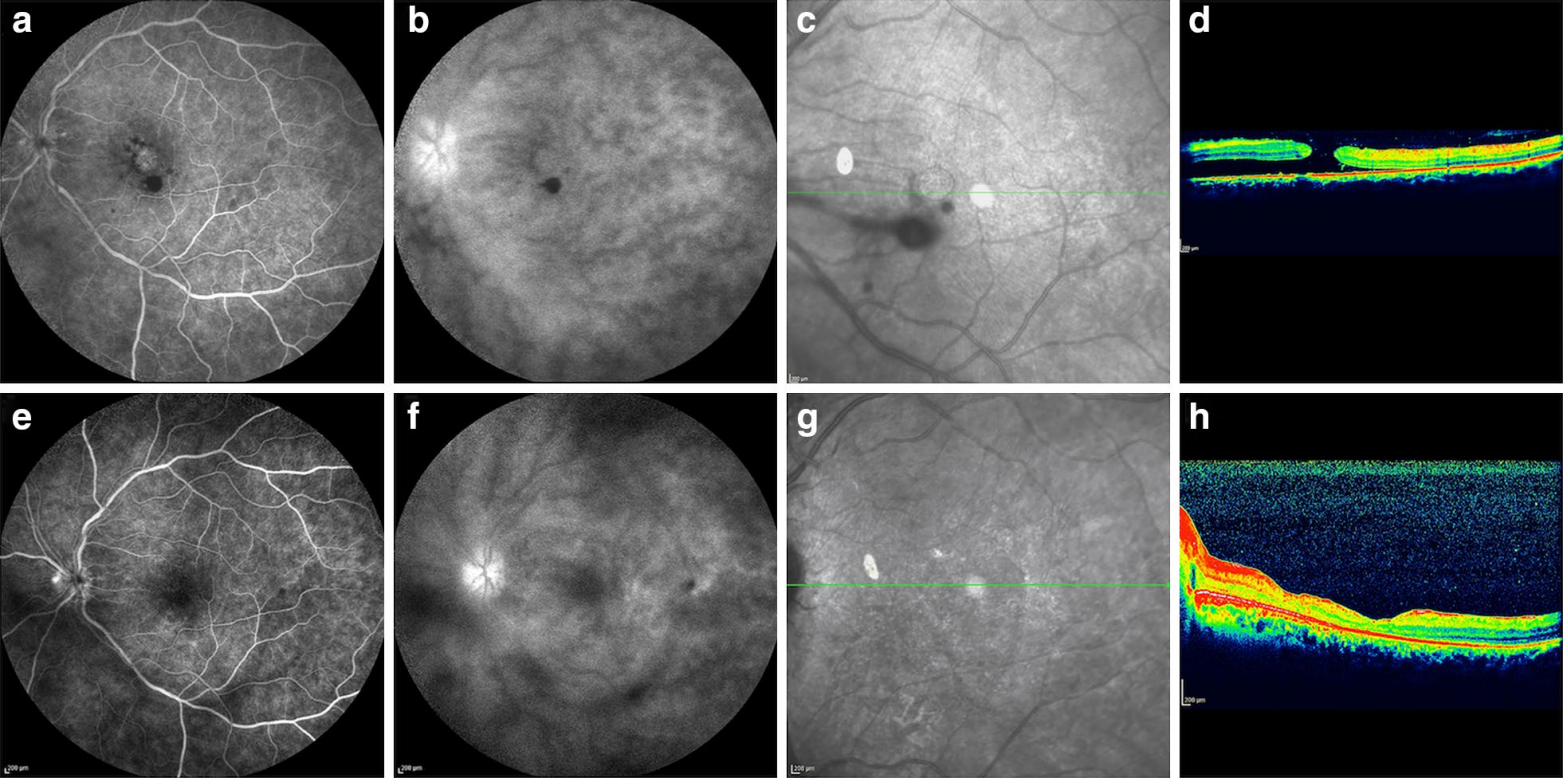
Fluorescein angiography (FA) and optical coherence tomography (OCT) images from a patient with a macular hole following acute uveitis in Vogt-Koyanagi-Harada syndrome. Preoperative (figures **a**–**d**) and postoperative images (**e–h**). **a** An early-phase FA image shows hyperfluorescence suggestive of retinal pigment epithelial (RPE) defects. **b** A late-phase FA image shows diffuse hyperfluorescence at the optic disc suggestive of persistent uveitis. **c** The position of the OCT scan at the macula. **d** An OCT image shows the macular hole (MH). A serous detachment of the macula is nasal to the fovea. **e** an early-phase postoperative FA image shows minimal hyperfluorescence suggestive of RPE defects. **f** A late-phase postoperative FA image shows diffuse hyperfluorescence at the optic disc suggestive of persistent uveitis less relevant than preoperatively. **g** The position of the OCT scan at the macula. **h** A postoperative OCT scan shows MH closure. No serous detachment of the macula is observed

**Fig. 2:**
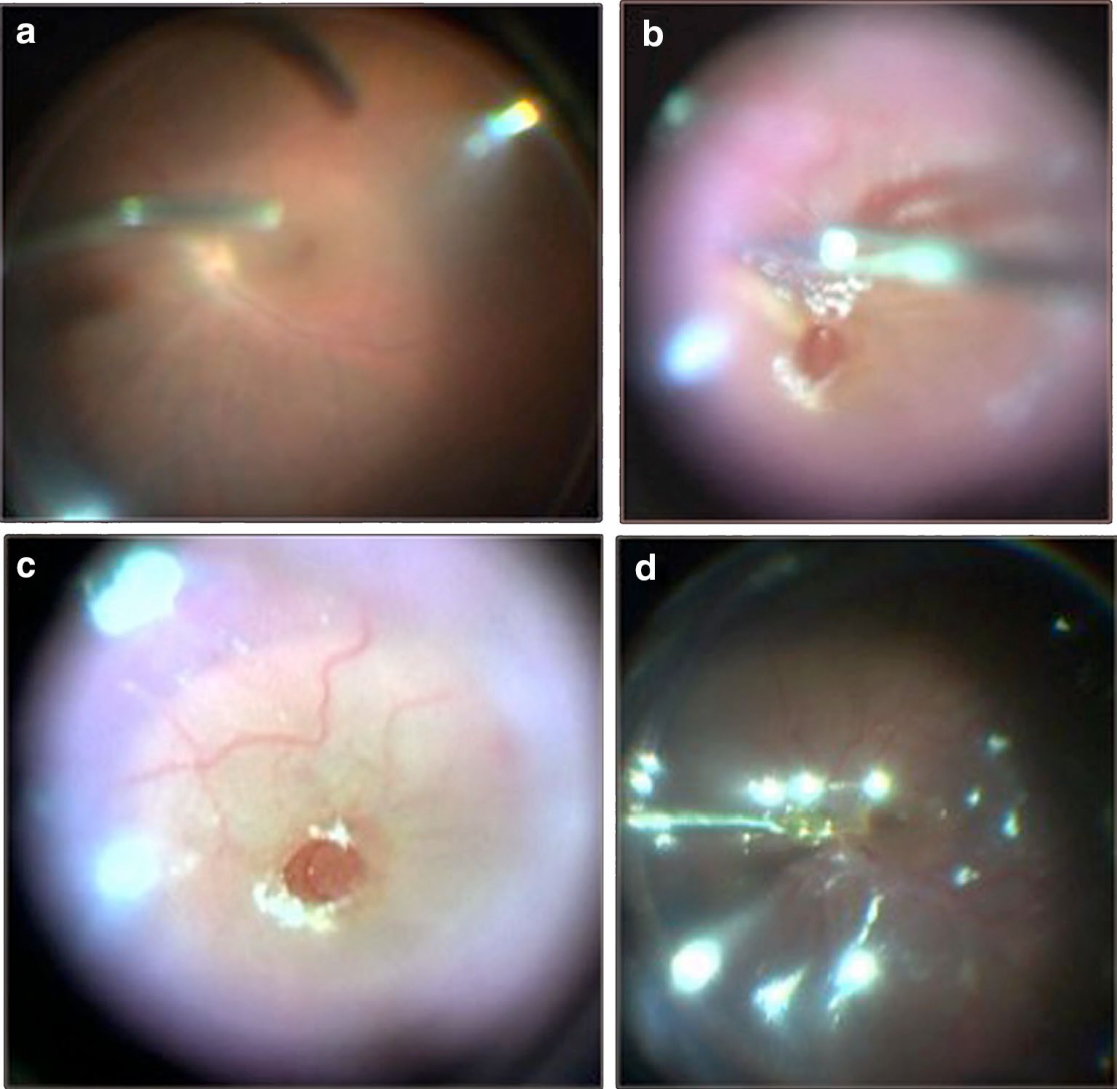
Macular hole surgery images. **a** Posterior hyaloid detachment guided by deposition of 40 mg/ml of triamcinolone acetonide and internal limiting membrane (ILM) peeling guided by injection of 0.50 mg/ml of brilliant blue. **b** ILM peeling after staining with Brilliant Blue. The ILM adheres tightly to the neurosensory retina. **c** The final stage of ILM peeling. The image shows the size of the MH and the peeled ILM around the MH. **d** Fluid-air exchange. Subretinal fluid resembling honey is drained through the MH that is likely related to subretinal fibrin collection due to the uveitis

Three days after this surgery, the patient reported worsening of the BCVA OS (counting fingers at 3 meters). Macular edema and papillitis OS were evident on examination. The patient underwent another course of a 3-day methylprednisolone 1 g/day pulse therapy, followed by 1.5 mg/kg/day of oral prednisone and 5 mg/kg/day of cyclosporine. The BCVA improved to 20/100 OS and the patient presented with mild anterior chamber flare and vitreous haze OU. After 7 days, optical coherence tomography (OCT) and fluorescein angiography (FA) showed residual edema of the optic disc, leakage at the optic disc and vessels, and an acute posterior vitreous detachment resulting in a MH OS (Fig. 1a–d) and a BCVA of 20/400. Because of the size of the MH (horizontal diameter, 824 micrometers; vertical diameter, 757 micrometers; maximum retinal thickness, 427 micrometers) and the absence of vitreous adherence seen on ultrasonography, we believed that spontaneous resolution was unlikely. Due to that and the poor BCVA in the fellow eye, it was decided to perform surgery promptly. The patient underwent a four-port PPV (three 23-gauge ports with a fourth 25-gauge chandelier light pipe). During surgery, it became evident that the posterior hyaloid was completely detached, by observing the deposition of triamcinolone acetonide 1 mg/ml (TA, Ophthalmos, SP, Brazil) at the retinal surface. ILM peeling was guided by 0.5 mg/ml of Brilliant Blue (Ophthalmos, SP, Brazil) staining (Fig. 2a–c) and it was observed to be tightly adhered to the neurosensory retina during peeling. A small macular detachment was present and fluid-air exchange was performed with aspiration of the subretinal thick fluid through the MH (Fig. 2d). Retinopexy was completed with 15 % C_3_F_8_, and prone positioning for 5 days postoperatively [4]. The BCVA improved to 20/40 OS. FA showed decreased hyperfluorescence at the foveal region (Fig. 1e, f), OCT showed resolution of the MH and a retinal thickness of about 183 microns (Fig. 1g, h) and the BCVA remained stable after at least 1 year postoperatively with titrating of topical steroids and oral prednisone (initially at 1 mg/kg/day).

## Discussion and conclusions

Chromovitrectomy assisted by posterior vitreous evaluation using TA and ILM peeling guided by Brilliant Blue 0.5 mg/ml staining followed by a 15 % C_3_F_8_ intravitreal injection and prone positioning of the patient for 5 days sealed the hole, reattached the macula and improved the BCVA from 20/400 preoperatively to 20/40 after the MH closed.

Postoperatively, the MH did not recur and no further inflammation developed during the 12-month follow-up period in which the patient continued therapy with prednisone 0.15 mg/kg/day and cyclosporine 5 mg/kg/day.

This case raised questions about the physiopathology of MH development. We hypothesized that the vitreous was previously attached to the posterior pole and tightly adhered to the macula and, after the onset of the inflammatory process, it acutely detached, resulting in a strong anteroposterior traction. This form of traction could be combined with a tangential traction by the ILM around the fovea, resulting in a large-diameter macular hole with irregular edges, similar to the physiopathogenesis of a traumatic macular hole. We also believe that this acute posterior vitreous detachment during an inflammatory surge could be linked to the vitreous hemorrhage and retinal detachment formerly observed in the right eye.

Two cases of VKH with a MH treated surgically have been reported; however, in those two cases the eyes were in the convalescent stage with no serous retinal detachment and had clinically relevant epiretinal membranes (ERM). The surgery included a standard three-port vitrectomy and peeling of the ERM and ILM. Fluid/air exchange was performed for air tamponade, and the patients were instructed to remain face-down for 3 days postoperatively [5]. The risk of inflammatory exacerbation in the present case with the surgery was weighed and explained to him and it was decided to carry on with the procedure, mainly due to his young age and the fact that the possibility of spontaneous closure of the macular hole was negligible with the concomitant macular detachment. Although some cases of closure without surgical intervention were reported, none presented with an associated retinal detachment [6].

Because ILM peeling associated with gas tamponade and the physical contact between the retinal pigment epithelium and the retina are considered important intraoperative factors for improving MH closure rates [7], we performed chromovitrectomy using TA to verify the status of the posterior hyaloids and Brilliant Blue 0.25 mg/ml for ILM staining and C_3_F_8_ injection into the vitreous cavity [4]. Because a serous macular detachment also was present, we drained the subretinal fluid, which resembled honey due to the exudative component of the subretinal fluid probably related to the inflammatory process. Despite the fact that drainage of subretinal fluid through the MH is contraindicated due to a poor prognosis [7], we did so after weighing the risks of inducing proliferative vitreoretinopathy related to an additional retinotomy in an inflamed eye versus no increased risks of such a complication if subretinal fluid is drained through the MH.

The outcomes of the current case, i.e., good anatomic and functional results, suggested that PPV may be an effective option for treating even large MH occurring during acute inflammatory stage of VKH disease. Additional studies are necessary to confirm the current findings.

## Abbreviations

VKH: Vogt-Koyanagi-Harada
MH: Macular hole
PPV: Pars plana vitrectomy
ILM: Internal limiting membrane
C_3_F_8_: Octafluoropropane
BCVA: Best-corrected visual acuity
OD: Right eye
OS: Left eye
OCT: Optical coherence tomography
FA: Fluorescein angiography
TA: Triamcinolone acetonide
ERM: Epiretinal membrane
